# Women with Acute Myocardial Infarction: Clinical Characteristics, Treatment, and In-Hospital Outcomes from a Latin American Country

**DOI:** 10.5334/gh.1196

**Published:** 2023-04-20

**Authors:** Alexandra Arias-Mendoza, Héctor González-Pacheco, Amada Álvarez-Sangabriel, Rodrigo Gopar-Nieto, Laura Leticia Rodríguez-Chávez, Diego Araiza-Garaygordobil, Pamela Ramírez-Rangel, Daniel Sierra-Lara Martínez, María del Carmen Lacy-Niebla, José Luis Briseño-De la Cruz, Jessica Juárez-Tolen, Salvador Mendoza-García, Alfredo Altamirano-Castillo

**Affiliations:** 1 Coronary Care Unit, Instituto Nacional de Cardiología Ignacio Chávez, Mexico City, Mexico; 2 Clinical Cardiology Department, Instituto Nacional de Cardiología Ignacio Chávez, Mexico City, Mexico

**Keywords:** women, acute coronary syndromes, treatment, diagnosis, gender differences

## Abstract

**Background:**

Women are underrepresented in acute myocardial infarction (AMI) studies. Furthermore, there is scarce information regarding women with AMI in Latin America.

**Aims:**

To describe the presentation, clinical characteristics, risk factor burden, evidence-based care, and in-hospital outcome in a population of women with AMI admitted to a coronary care unit (CCU) in Mexico.

**Methods:**

Retrospective cohort study including patients with AMI admitted from January 2006 to December 2021 in a CCU. We identified patients with ST-segment elevation myocardial infarction (STEMI) and non-ST-segment elevation myocardial infarction (NSTEMI). We described demographic characteristics, clinical variables, treatment, and in-hospital outcomes according to gender. Cox regression analysis was used to identify predictors of mortality.

**Results:**

Our study included 12,069 patients with AMI, of whom 7,599 had STEMI and 4,470 had NSTEMI. Women represented 19.6% of the population. Women had higher rates of hypertension, diabetes, stroke, and atrial fibrillation than men. For STEMI, women were less likely to receive reperfusion therapy (fibrinolysis; 23.7 vs. 28.5%, p < 0.001 and primary percutaneous coronary intervention (PCI); 31.2 vs. 35.1%, p = 0.001) and had more major adverse events than men: heart failure (4.2 vs. 2.5%, p = 0.002), pulmonary edema (3.4% vs. 1.7%, p < 0.001), major bleeding (2.1% vs. 1%, p = 0.002), stroke (1.3% vs. 0.6%, p = 0.008), and mortality (15.1% vs. 8.1%, p < 0.001). For NSTEMI, women were less likely to undergo coronary angiography or PCI and had more major bleeding and mortality. Multivariate Cox regression analysis revealed that females had an increase in mortality in STEMI and NSTEMI (HR 1.21, CI 1.01–1.47, p = 0.05 and HR 1.39, CI 1.06–1.81, p = 0.01).

**Conclusion:**

Real-world evidence from a hospital in a Latin American low- to middle-income country (LMIC) showed that women with AMI had more comorbidities, received less reperfusion treatment or invasive strategies, and had worse outcomes. In STEMI and NSTEMI, female gender represented an independent predictor of in-hospital mortality.

## Introduction

Ischemic heart disease remains the leading cause of mortality and is a public health challenge worldwide [1]. In the setting of acute myocardial infarction (AMI), despite advancements in treatment during the last decades, women continue to have worse short- and long-term outcomes than men [2,3]. Several studies have revealed sex-specific differences in AMI regarding clinical presentation, pathophysiological mechanisms, diagnosis, management, and outcomes [4,5,6,7,8,9,10]. Nevertheless, women remain understudied, as they only represent about 20% of the patients enrolled in AMI clinical trials [7].

Ethnicity, socioeconomic status, and gender are important considerations in the prevalence of treatment and outcomes of AMI worldwide. There is evidence that in LMICs, AMI prognosis in women is modified according to many factors, including socioeconomic background [8]. Furthermore, the prevalence of myocardial infarction and outcomes in black women are worse compared to other groups; however, other ethnic groups have not been studied in depth [11,12].

Since there is scarce information for Latin American women, the aim of our study was to describe the presentation, clinical characteristics, risk factor burden, evidence-based care, and in-hospital outcomes in a population of women with AMI admitted to a coronary care unit (CCU) in an LMIC in Latin America.

## Materials & Methods

### Methods

This was a retrospective cohort study that used data from the database of the CCU of the National Institute of Cardiology in Mexico City, which is a cardiovascular tertiary referral university center. We analyzed all patients admitted between January 1, 2006, and December 31, 2021, with AMI diagnosis and classified as having either an ST-segment elevation myocardial infarction (STEMI) or non ST-segment elevation myocardial infarction (NSTEMI) based on clinical characteristics, electrocardiographic changes, and biochemical markers of cardiac necrosis (isoenzyme of creatinine kinase, creatinine phosphokinase, or troponin I), according to the standard definitions of the American College of Cardiology and the European Society of Cardiology.

We analyzed demographic data, medical history, clinical presentation, laboratory tests, coronary angiography, management during hospital stay (including medications, reperfusion therapy, and procedures), and in-hospital outcomes.

The diagnosis of AMI was based on clinical characteristics, electrocardiographic changes, and blood levels of biochemical markers of cardiac necrosis (creatinine kinase isoenzymes, creatinine phosphokinase, troponin I or troponin T), and it was classified as STEMI or NSTEMI according to the American College of Cardiology criteria. Creatinine clearance was estimated using the Cockcroft-Gault formula. To establish the diagnosis of heart failure, we considered the following: a history of progressive worsening functional class with treatment and evidence of systemic and pulmonary congestion, severe respiratory distress, orthopnea, crackles over the lungs, and NTproBNP elevation. To diagnose cardiogenic shock, we used the criteria of systolic blood pressure <90 mmHg for ≥30 min or the need for catecholamines to maintain systolic blood pressure >90 mmHg, clinical pulmonary congestion, and organ hypoperfusion with any of the following symptoms: cold extremities, confusion or altered mental status, oliguria, or blood lactate >2.0 mmol/L. All the manuscript was based on the STROBE guidelines.

### Statistical analysis

For analytical purposes, women were compared with men admitted with AMI diagnosis during the same period. Categorical data were summarized as frequencies and percentages. All continuous variables were tested and confirmed to have a nonnormal distribution as determined by the Kolmogorov-Smirnov test. Continuous variables were reported as medians and 25th and 75th percentiles (interquartile ranges, IQRs). Statistically significant differences between groups were assessed using either a Chi-square or Fisher’s exact probability test for categorical variables or the Mann-Whitney U test for continuous variables. The study outcomes were defined as all-cause in-hospital mortality. Unadjusted Cox proportional hazards regression models were performed to estimate the risk of in-hospital mortality. Multivariate Cox’s proportional hazards regression models with backward selection were used to adjust for the potential confounding on the basis of the established associations between women and all-cause in-hospital mortality. The candidate covariates were those associated with mortality in a univariate analysis, which included all of the baseline characteristics, clinical features on presentation, and the absence of reperfusion therapy that had p < 0.05, as well as those recognized as prognostic factors based on previous medical knowledge. Separate regression models were estimated for STEMI and NSTEMI. The hazard ratios (HR) with 95% confidence intervals (CI) were calculated. Results are reported in terms of their two-tailed significance. IBM SPSS Statistics for Windows (v. 23; IBM Corp., Armonk, NY, USA) was used for all analyses.

## Results

**Figure 1:**
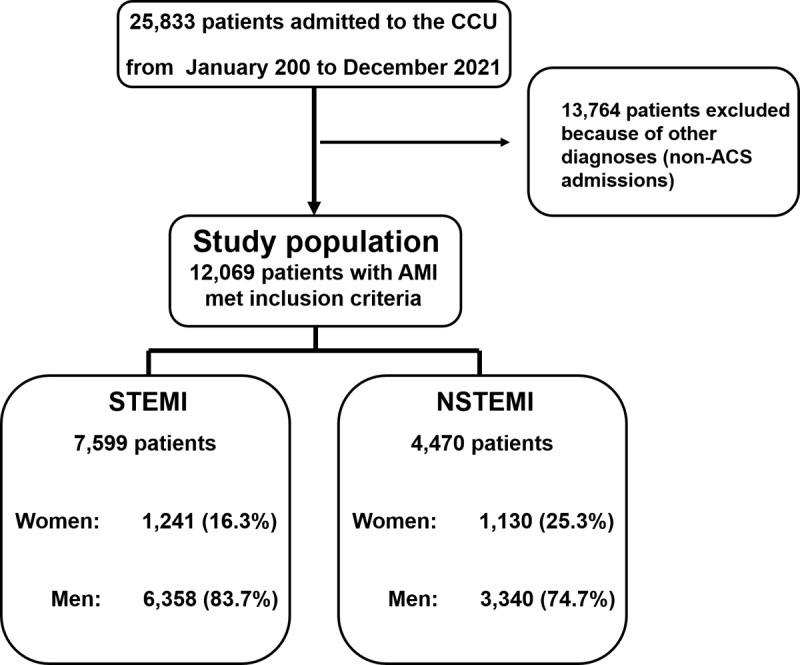
Flow chart illustrating the study sample selection.

During the study period, a total of 12,069 patients with acute coronary syndrome (ACS) were admitted to our center (7,599 with STEMI and 4,470 with NSTEMI) and constituted the final analytical sample, as seen in the study flow chart (Figure 1). Women represented 19.6% of the population (n = 2,371) and we detected differences between STEMI and NSTEMI (16.3% vs. 25.3%).

Descriptions of the baseline characteristics of the study population are shown in Table 1. Women were older than men in both STEMI (65 years vs. 58 years, p < 0.0001) and NSTEMI (67 years vs. 62 years, p < 0.0001). Men had higher body mass index, prior myocardial infarction, and dyslipidemia than women, but women had strikingly higher rates of hypertension and diabetes as well as more prior stroke and atrial fibrillation (AF).

**Table 1:** Baseline characteristics of patients with STEMI and NSTEMI.


	STEMI			NSTEMI		
	
	WOMEN (N = 1,241)	MEN (N = 6,358)	*P*-VALUE	WOMEN (N = 1,130)	MEN (N = 3,340)	*P*-VALUE

**Age (years)Median (IQR), (n = 12,051)**	65 (57–74)	58 (51–65)	<0.0001	67 (60–75)	62 (54–70)	<0.0001

**BMI (kg/m^2^)Median (IQR), (n = 12,050)**	26.6 (23.9–29.4)	27.2 (24.9–29.4)	<0.0001	26.6 (24–29.4)	26.9 (24.8–29.7)	<0.0001

**Current/recent smoker, n (%)**	239 (19.3)	2,381 (37.4)	<0.0001	128 (11.3)	784 (23.5)	<0.0001

**Hypertension, n (%)**	818 (65.9)	2,842 (44.7)	<0.0001	886 (78.4)	2,080 (62.3)	<0.0001

**Dyslipidemia, n (%)**	349 (28.1)	1,850 (29.1)	0.25	470 (41.6)	1,523 (45.6)	0.20

**Diabetes, n (%)**	638 (51.4)	2,254 (35.5)	<0.0001	598 (52.9)	1,333 (39.9)	<0.0001

**Prior myocardial infarction, n (%)**	110 (8.9)	903 (14.2)	<0.0001	389 (34.4)	1,562 (46.8)	<0.0001

**Prior PCI, n (%)**	56 (4.5)	509 (8.0)	<0.0001	189 (16.7)	839 (25.1)	<0.0001

**Prior CABG, n (%)**	12 (1.0)	70 (1.1)	0.40	66 (5.8)	272 (8.1)	0.01

**Prior stroke, n (%)**	38 (3.1)	119 (1.9)	<0.0001	47 (4.2)	99 (3.0)	0.05

**Prior heart failure, n (%)**	43 (3.5)	210 (3.3)	0.73	208 (18.4)	535 (16.0)	0.06

**Atrial fibrillation, n (%)**	32 (2.6)	48 (0.8)	<0.0001	73 (6.5)	97 (2.9)	<0.0001

**Prior use of aspirin, n (%)**	228 (18.4)	1,160 (18.2)	0.90	564 (49.9)	1,798 (53.8)	0.02

**Prior use of P2Y12 receptor inhibitors, n (%)**	48 (3.9)	289 (4.5)	0.28	163 (14.4)	564 (16.9)	0.05

**Prior use of beta blockers, n (%)**	210 (16.9)	756 (11.9)	<0.0001	447 (39.6)	1,271 (38.1)	0.37

**Prior use of statin, n (%)**	156 (12.6)	918 (14.1)	0.09	529 (46.8)	1,718 (51.4)	<0.0001

**Prior use of ACEI/ARB (%), n (%)**	574 (46.3)	2,011 (31.6)	<0.0001	702 (62.1)	1,781(53.3)	<0.0001


STEMI: ST-segment elevation myocardial infarction; NSTEMI: non-ST-elevation myocardial infarction; IQR: interquartile range; BMI: body mass index; PCI: percutaneous coronary intervention; CABG: coronary artery bypass grafting; ACEI/ARB: angiotensin-converting enzyme inhibitors/angiotensin receptor blocker.

Regarding the use of medications before hospital admission, women with NSTEMI received less aspirin, statins, and P2Y12 receptor inhibitors. In STEMI, women received more beta blockers and angiotensin-converting enzyme inhibitors/angiotensin receptor blockers than men (Table 1).

On admission, the patients with AF had high-risk clinical characteristics and developed major adverse events more frequently than did the patients without AF, and women showed more pulmonary congestion, as shown by a higher Killip and Kimbal class, both in STEMI and in NSTEMI (Table 2). Time from symptom onset to arrival for medical care was significantly higher in women with STEMI (14:17 hh:mm vs. 10:56 hh:mm, p < 0.001), but it was not significantly higher in women with NSTEMI (9:24 hours vs. 8:52 hours, p = 0.32). Women had higher blood glucose in both ACS categories, as well as lower hemoglobin. Creatinine clearance categorization showed that women had lower filtration rates than men both in STEMI and NSTEMI (Table 2).

**Table 2:** Characteristics at admission.


		STEMI			NSTEMI		
	
		WOMEN (N = 1,241)	MEN (N = 6,358)	*P*-VALUE	WOMEN (N = 1,130)	MEN (N = 3,340)	*P*-VALUE

**Systolic blood pressure (mmHg)Median (IQR), (n = 12,044)**		125 (110–145)	128 (110–138)	0.04	132 (120–150)	130 (120–150)	<0.0001

**Heart rate (beats/min)Median (IQR), (n = 12,040)**		80 (70–92)	80 (70–91)	0.56	79 (70–90)	76 (68–89)	<0.0001

**Killip class II–IVn (%)**		619 (49.9)	2,430 (38.2)	<0.0001	429 (38)	929 (27.8)	<0.0001

**Anterior wall location n (%)**		558 (45)	3100 (48.8)	0.01	–	–	–

**Time from symptom onset to arrival (hours)Median (IQR), (n = 11,972)**		14:17 (5:18–45:33)	10:56 (4:09–36:38)	<0.0001	9:24(4:00–24:49)	8:52 (3:48–23:30)	0.32

**LVEF (%)Median (IQR), (n = 11,378)**		50 (40–55)	50 (40–55)	0.20	53 (42–60)	51 (40–58)	<0.0001

**Admission blood glucose level (mg/dL)Median (IQR), (n = 12,004)**		159 (119–233)	143 (114–207)	<0.0001	138 (106–211)	123 (101–173)	<0.0001

**Hemoglobin (g/L)Median (IQR), (n = 12,045)**		13.2 (12–14.4)	15.4 (14–16.5)	<0.0001	13.0 (11.6–14.2)	15.0 (13.6–16)	<0.0001

**Creatinine (mg/dL)Median (IQR), (n = 12,046)**		1.0 (0.8–1.4)	1.0 (0.7–1.2)	0.30	0.9 (0.7–1.3)	1.0 (0.9–1.3)	0.15

**Creatinine clearance(mg/dL)n (%)**	<30 mL/h	151 (12.2)	326 (5.1)	<0.0001	168 (14.9)	246 (7.4)	<0.0001
	
	30–59 mL/h	385 (31.0)	979 (15.4)		399 (35.3)	732 (21.9)	
	
	>60 mL/h	705 (56.8)	5,053 (79.5)		563 (49.8)	2,362 (70.7)	


STEMI: ST-segment elevation myocardial infarction; NSTEMI: non-ST-elevation myocardial infarction; IQR: interquartile range; LVEF: left ventricular ejection fraction.

In STEMI, women received fewer overall reperfusion therapies (54.8% vs. 63.7%, p < 0.0001). Primary percutaneous coronary intervention (PCI) was performed more on men as well as out-of-hospital thrombolysis, while the percentage of thrombolysis in our hospital was very similar. In NSTEMI, women were less likely to receive coronary angiography (70.7% vs. 78.6%, p < 0.0001) and PCI (36.7% vs. 48.5%, p < 0.0001) as compared to men (Table 3). There was no difference among women and men in the proportion receiving aspirin, heparin, and statin in either of the AMI groups. Nevertheless, in both STEMI and NSTEMI groups, women were significantly less likely to receive beta blockers, angiotensin-converting enzyme inhibitors/angiotensin receptor blockers, and P2Y12 receptor inhibitors (Table 3).

**Table 3:** In-hospital management.


	STEMI			NSTEMI		
	
	WOMEN (N = 1,241)	MEN (N = 6,358)	*P*-VALUE	WOMEN (N = 1,130)	MEN (N = 3,340)	*P*-VALUE

**Reperfusion therapy, n (%)**	680 (54.8)	4,047(63.7)	<0.0001			

**—Primary PCI, n (%)**	387 (31.2)	2,233 (35.1)	<0.0001			

**—In-hospital thrombolysis, n (%)**	90 (7.3)	524 (8.2)	<0.0001			

**—Out-of-hospital thrombolysis, n (%)**	203 (16.4)	1,290 (20.3)	<0.0001			

**Coronary angiography, n (%)**	1,002 (80.7)	5,575 (87.7)	<0.0001	799 (70.7)	2,626 (78.6)	<0.0001

**No primary PCI, n (%)**	485 (39.1)	2,673 (42.0)	0.05	415 (36.7)	1,621 (48.5)	<0.0001

**Aspirin, n (%)**	1,224 (98.6)	6,302 (99.1)	0.10	1,115 (98.7)	3,297 (98.7)	0.91

**P2Y12 receptor inhibitors, n (%)**	1,044 (84.1)	5,681 (89.4)	<0.0001	813 (71.9)	2,539 (76.0)	0.006

**Heparin, n (%)**	1,215 (97.9)	6,276 (98.7)	0.02	1,107 (98.0)	3,270 (97.9)	0.90

**Statin, n (%)**	1,212 (97.7)	6,248 (98.)	0.14	1,101 (97.4)	3,258 (97.5)	0.83

**Beta blockers, n (%)**	617 (49.7)	3,941 (62.0)	<0.0001	726 (64.2)	2,308 (69.1)	0.003

**ACEI/ARB (%), n (%)**	997 (80.)	5,530 (87.0)	<0.0001	972 (86.0)	2,957 (88.5)	0.02


STEMI: ST-elevation myocardial infarction; NSTEMI: non-ST-elevation myocardial infarction; PCI: percutaneous coronary intervention; ACEI/ARB: angiotensin-converting enzyme inhibitors/angiotensin receptor blocker.

**Figure 2:**
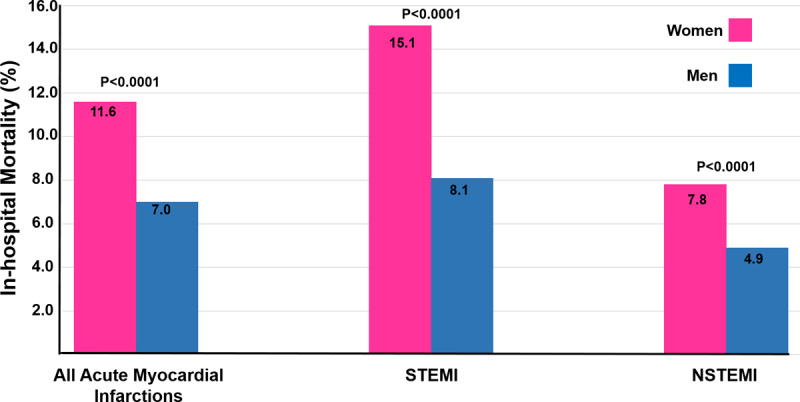
Overall in-hospital all-cause mortality rates of the 12,069 CCU admissions with acute myocardial infarction. In-hospital all-cause mortality rates among 7,599 patients with ST-elevation myocardial infarction and 4,470 patients with non-ST-elevation myocardial infarction.

**Figure 3:**
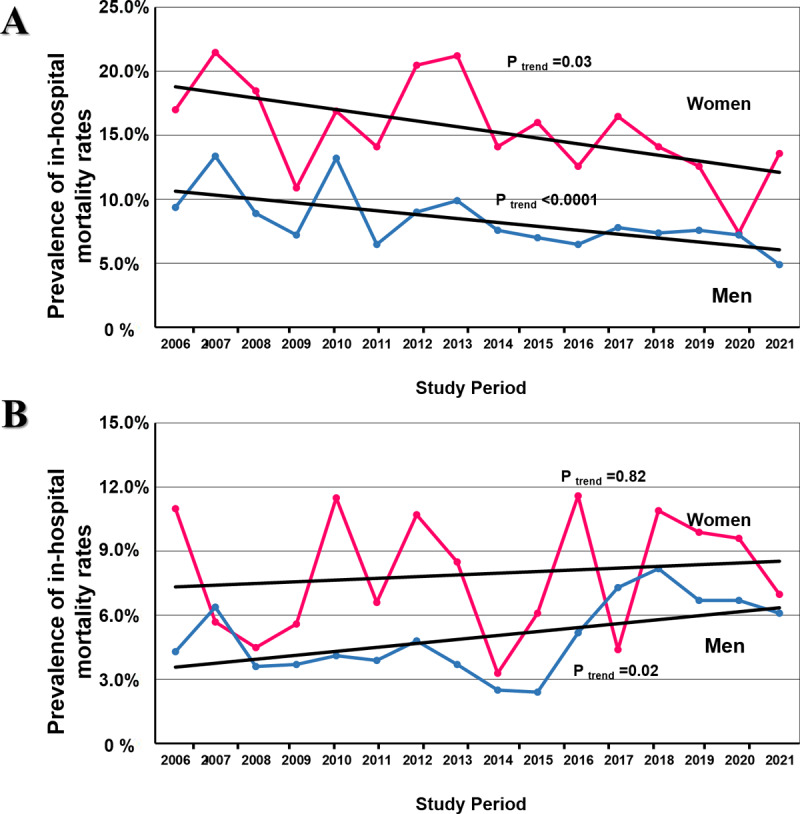
Temporal trends from 2006 to 2021 in rates of all-cause in-hospital mortality: **(A)** In patients with ST-elevation myocardial infarction. **(B)** In patients with non-ST-elevation myocardial infarction.

In the whole cohort, the unadjusted in-hospital mortality rates were significantly higher in women compared to men (11.6% vs. 7.0%, p < 0.0001). In both the STEMI and NSTEMI patient groups, the unadjusted all-cause in-hospital mortality rate was higher in women (15.1% vs. 8.1% and 7.8% vs. 4.9%, respectively, p < 0.0001) (Figure 2). In the period analyzed (2006–2021), in the STEMI group, the mortality rate in women had statistically significant downward trend over time (17.3% to 13.6%, P_trend_ = 0.03) (Figure 3-A). Similarly, in men, the same trend was observed over time (9.4% to 4.9%, P_trend_ < 0.0001). However, in the NSTEMI group, mortality in women remained unchanged during the studied period (11.3% to 7.0%, P_trend_ < 0.82), while in men there was an upward trend (4.3% to 6.1%, P_trend_ < 0.02) (Figure 3-B).

**Figure 4:**
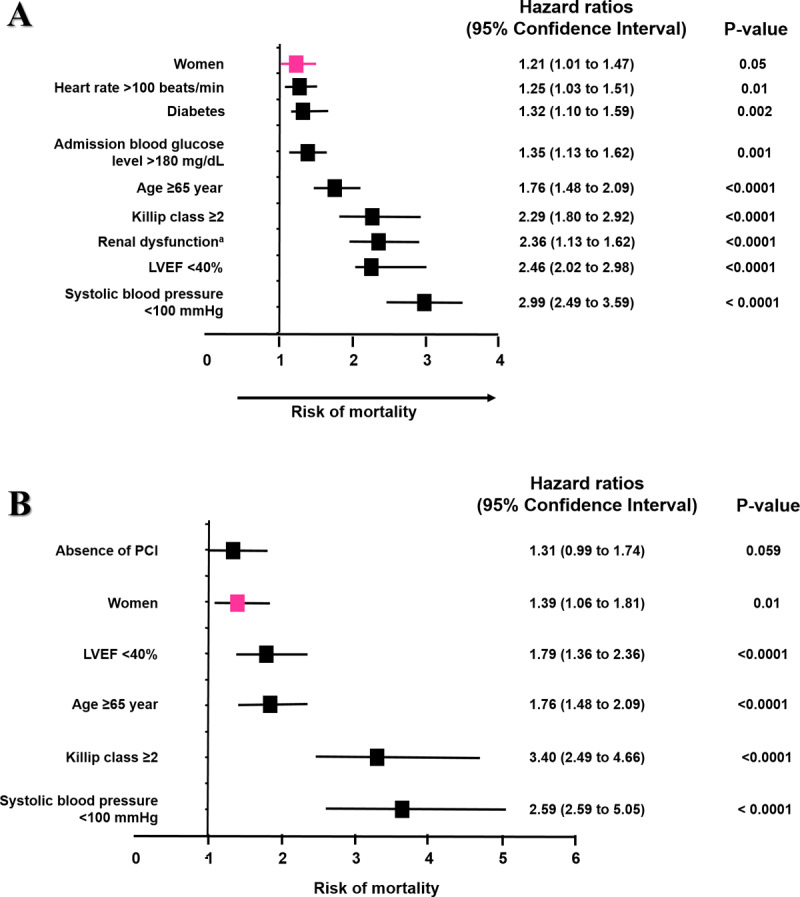
Cox regression model: **(A)** Independent predictors of in-hospital all-cause mortality in patients with ST-elevation myocardial infarction. **(B)** Independent predictors of in-hospital all-cause mortality in patients with non-ST-elevation myocardial infarction.

In both STEMI and NSTEMI groups, the unadjusted analysis showed that women had increased risk of mortality (HR of 1.72, 95% CI 1.45–2.03, p < 0.0001; HR of 1.64, 95% CI 1.27–2.13, respectively, p < 0.0001). Adjusted multivariate Cox proportional hazards regression models were generated separately for STEMI and NSTEMI with all statistically significant univariate predictors of in-hospital mortality at admission, listed in Tables 1 and 2. The analysis demonstrated that female gender in the STEMI group was associated with a 1.21-fold increase in the risk of in-hospital mortality (HR 1.21, CI 1.01–1.47, p = 0.05) (Figure 4-A). Likewise, women in the NSTEMI group were associated with an increase in the in-hospital mortality risk (HR 1.39, CI 1.06–1.81, p = 0.01) (Figure 4-B).

**Figure 5:**
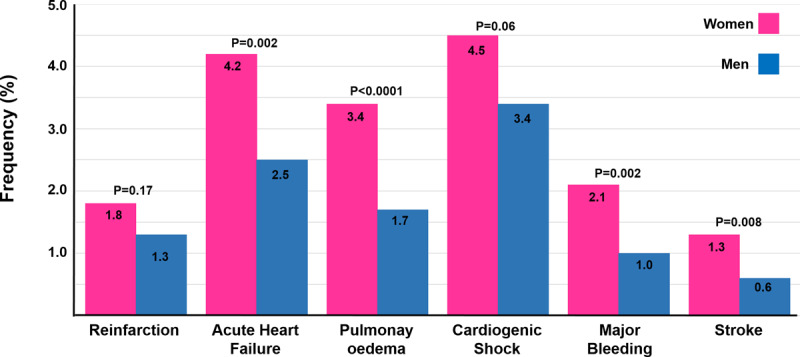
In-hospital adverse events in patients with ST-elevation myocardial infarction.

In addition, in-hospital outcomes for STEMI showed that women had more acute heart failure (4.2% vs. 2.5%, p = 0.002), pulmonary edema (3.4% vs. 1.7%, p < 0.0001), major bleeding (2.1% vs. 1%, p = 0.002), and stroke (1.3% vs. 0.6%, p = 0.008), while there were no significant differences for cardiogenic shock (4.5% vs. 3.4%, p = 0.06) (Figure 5). Meanwhile, for NSTEMI, women had more major bleeding (0.9% vs. 0.6%, p = 0.03), while there were no significant differences for reinfarction, acute heart failure, pulmonary edema, stroke, and cardiogenic shock.

## Discussion

In our study, we found that women had a higher prevalence of cardiovascular risk factors and a more severe clinical presentation, received less treatment, and showed worse in-hospital outcomes, mainly represented by mortality, in STEMI and NSTEMI, as compared to men.

Women with ACS have more comorbidities than men, as we describe in our study: they are older and have a higher proportion of hypertension, diabetes, and atrial fibrillation than men, besides having a higher risk profile because they more commonly had higher Killip-Kimball class, glucose levels, and renal dysfunction. These characteristics have been described in other studies [2,13,14,15,16,17,18], but we can add that Mexican ancestry shows a particular genetic background that makes them more susceptible to metabolic diseases [19], which may contribute to worse outcomes. Furthermore, we must also consider the social and economic disparities in our population, which are represented by the patients that do not have access to social security services in Mexico, that contribute to the appearance of ACS and adverse outcomes that have been described in the literature [7,8].

Women receive fewer coronary angiographies and reperfusion treatments than men in ACS, as was proven in our study. Even though early reperfusion can save more myocardium and reperfusion strategies such as pharmacoinvasive strategy and primary PCI are widely available, this trend has not been equally distributed in both genders and can even extend to young women [13]. Therefore, strategies must be implemented for women to have more reperfusion and invasive strategies to treat ACS.

Our findings agree that mortality is higher in women with ACS admitted to CCUs, as has been previously described [2,16,17,18]; nevertheless, our results differ from those found in Spanish population, in which women had lower mortality in NSTEMI [20] and we found increased risk of in-hospital mortality both in STEMI and NSTEMI. Coronary heart disease has traditionally been described as a men’s disease, and the symptoms that are considered ‘typical’ are more frequent in men [7]. Women may have different initial symptoms, such as dizziness, nausea, and, less frequently, chest pain; one of the main reasons for the increment in mortality rates among women is the bias in the recognition of the initial symptoms not only in the primary medical contact but also by the patient herself [21]. We must emphasize that the main determinant of favorable outcomes in STEMI is the time to reperfusion [22,23], and in our study, we found that women arrived later than men at the emergency department after the onset of symptoms; therefore, an increase in mortality and adverse outcomes were expected in this group.

Regarding other outcomes, we were able to describe that women have more heart failure, pulmonary edema, major bleeding, and stroke, with a marginal increase in cardiogenic shock in STEMI, while these differences blunted with NSTEMI and persisted only for major bleeding and mortality. These factors may be explained from many perspectives, but biologically they may be caused by different responses to drugs that are administered for ACS, hormonal differences, and body composition [2,3,4]. Along with the biological explanations, socioeconomic and demographic factors might contribute to the presence of adverse outcomes [7,8] because of education, transport, or medical services.

Our study represents a contribution to the lack of evidence in LMICs in Latin America regarding clinical characteristics and outcomes in women with ACS. Evidence-based decisions cannot be made if there is no data available for clinicians to compare with their native populations.

### Limitations

Our study has several limitations. First, we used retrospective data from a single hospital in Mexico City. Second, we must highlight that our hospital is a referral center that receives patients from all of Mexico; nevertheless, the reperfusion trends and therapeutics may be different within our own country. Finally, our study only included patients who were hospitalized in the CCU rather than including those hospitalized in internal wards, as was the case in other reports.

## Conclusion

There are substantial differences in AMI demographics, treatment, and outcomes according to gender. Women have more baseline comorbidities, higher risk conditions at admission, and receive less reperfusion treatments or invasive strategies, with the consequences of worse outcomes. Our results showed that in-hospital mortality is substantially increased for women with STEMI and NSTEMI. There is an urgent need for the creation of evidence regarding gender differences in AMI in Latin America and other low- to middle-income regions.
